# Photodetection Properties of MoS_2_, WS_2_ and Mo_x_W_1-x_S_2_ Heterostructure: A Comparative Study

**DOI:** 10.3390/nano13010024

**Published:** 2022-12-21

**Authors:** Maryam Al Qaydi, Ahmed Kotbi, Nitul S. Rajput, Abdellatif Bouchalkha, Mimoun El Marssi, Guillaume Matras, Chaouki Kasmi, Mustapha Jouiad

**Affiliations:** 1 Laboratory of Physics of Condensed Mater, University of Picardie Jules Verne, 80039 Amiens, France; 2 Technology Innovation Institute, Abu Dhabi P.O. Box 9639, United Arab Emirates

**Keywords:** photodetection, 2D materials, MoS_2_, WS_2_, Mo_x_W_1-x_S_2_ heterostructure, CVD growth

## Abstract

Layered transition metals dichalcogenides such as MoS_2_ and WS_2_ have shown a tunable bandgap, making them highly desirable for optoelectronic applications. Here, we report on one-step chemical vapor deposited MoS_2_, WS_2_ and Mo_x_W_1-x_S_2_ heterostructures incorporated into photoconductive devices to be examined and compared in view of their use as potential photodetectors. Vertically aligned MoS_2_ nanosheets and horizontally stacked WS_2_ layers, and their heterostructure form Mo_x_W_1-x_S_2_, exhibit direct and indirect bandgap, respectively. To analyze these structures, various characterization methods were used to elucidate their properties including Raman spectroscopy, X-ray diffraction, X-ray photoelectron spectrometry and high-resolution transmission electron microscopy. While all the investigated samples show a photoresponse in a broad wavelength range between 400 nm and 700 nm, the vertical MoS_2_ nanosheets sample exhibits the highest performances at a low bias voltage of 5 V. Our findings demonstrate a responsivity and a specific detectivity of 47.4 mA W^−1^ and 1.4 × 10^11^ Jones, respectively, achieved by Mo_x_W_1-x_S_2_. This study offers insights into the use of a facile elaboration technique for tuning the performance of Mo_x_W_1-x_S_2_ heterostructure-based photodetectors.

## 1. Introduction

Semiconductor photodetectors, namely photodiodes, are the most common types of detectors used in optical communication systems owing to their compact size, fast detection speed and high detection efficiency. Practical photodiodes can have a variety of device structures, beyond the basic PN junction construction, to improve their efficiency [1,2]. High-performance photodetectors have been used in a wide range of applications, including electro-optical displays [3], imaging [4], environmental monitoring [5], optical communication [6], military applications and security checks [7]. In these domains, two-dimensional (2D) materials, especially transition-metal dichalcogenides (TMDs), are becoming more attractive for designing photodetectors [8,9,10] due to their unique properties such as their ability to operate in the full range of visible light while having high photodetection polarization sensitivity, a fast photoresponse and high spatially resolved imaging [9]. This class of materials exhibits a layer-dependent electronic band structure in terms of unique physical characteristics and detection mechanisms [11,12]. Photodetectors based on 2D-TMDs materials are more sensitive throughout a wide range of the electromagnetic spectrum compared to photodetectors based on conventional bulk semiconductors [8]. However, an enhanced absorption coefficient and a longer lifespan of photoexcited carriers are preferred for optimal photocurrent generation and photodetector operation. For instance, MoS_2_ thin films have a high light absorption coefficient of 10^7^ m^−1^, a high absorption of 95% of total light [13] and a direct bandgap of 1.8 eV. Moreover, photodetectors based on 2D-TMDs possess a good current on/off ratio, efficiency, higher chemical and mechanical stability and a stronger light–matter interaction compared to conventional photodetectors. Furthermore, mono- and few-layers MoS_2_ present other desirable electronic properties that make them suitable for optoelectronic applications, such as their high carrier mobility and electrostatic integrity [14,15,16]. Owing to these properties, a MoS_2_ single-layered transistor was found to achieve a responsivity up to 880 × 10^3^ mA W^−1^ under a 560 nm excitation [17]. Moreover, an ultrabroadband multilayer MoS_2_ photodetector was reported to operate in 445–2717 nm range achieving a responsivity and a specific detectivity of 50.7 mA W^−1^ and 1.6 × 10^9^ Jones, respectively [18]. It was also shown that the photoresponse of the MoS_2_ photodetector could be enhanced by chemical doping to improve its responsivity and specific detectivity up to 10^5^ mA W^−1^ and 9.4 × 10^12^ Jones, respectively [19]. The reported responsivity and specific detectivity values are approximately 15 and 5 times higher relative to those of pristine photodetector. In addition to that, WS_2_ is considered as another promising candidate for photodetection owing to a range of outstanding properties, such as its bandgap tunability, its high carrier mobility and its efficient optical absorption [20,21]. WS_2_-based photodetectors have been reported to exhibit a responsivity of 4 mA W^−1^ at operating wavelengths ranging from the visible to the near infrared (IR) range [22]. Once combined with other 2D-materials such as graphene, the resulting heterostructure has shown a higher responsivity and specific detectivity of 3.5 × 10^3^ mA W^−1^ and 10^12^ Jones, respectively [23]. First attempts to use a Mo_x_W_1-x_S_2_ heterostructure as a photodetector showed promising performances such as a responsivity of 2.3 × 10^3^ mA W^−1^ obtained under 450 nm excitation [24], and a responsivity and a specific detectivity of 6.7 × 10^6^ mA W^−1^ and 3.1 × 10^13^ Jones under 457 nm laser light, respectively [25]. Other interesting works on alloying and ternary 2D-TMD materials can be found elsewhere [26,27,28].

In this work, a systematic comparative study is conducted on MoS_2_, WS_2_ and Mo_x_W_1-x_S_2_ heterostructure to emphasize their photodetection performances while using identical fabrication and analysis routes. All samples were fabricated in one single step chemical vapor deposition (CVD) process and underwent extensive characterization investigations.

## 2. Materials and Methods

**Figure 1:**
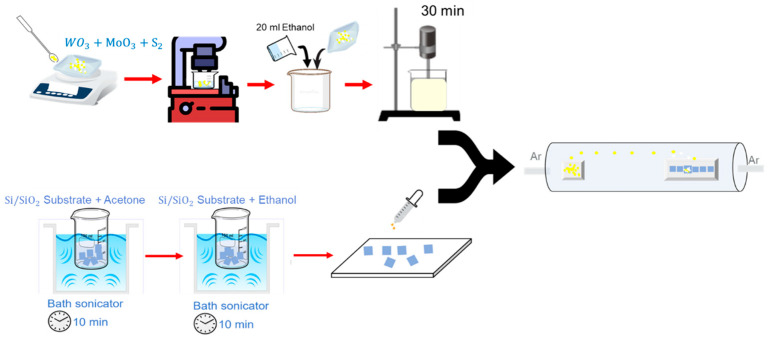
Fabrication protocol used for the MoS_2_, WS_2_ and Mo_x_W_1-x_S_2_ heterostructure samples.

Among several techniques used to fabricate 2D materials, CVD is the commonly employed technique to control defects, crystallinity, and morphology of this class of materials. In particular, CVD sulfurization process is a facile one-step processing route that allows the fabrication of several sulfur based 2D-materials. The fabrication control is often ensured by monitoring several processing parameters such as gas flow, temperature, heating rate, the distance between precursors, and the position and height of the collecting substrate [29]. The synthesis of all samples was obtained in a one-step CVD process using a single-heating zone furnace at atmospheric pressure, as shown in Figure 1. The CVD system mainly consists of a quartz tube connected to high-purity (99.999%) argon cylinder streaming at flow rates of 70 sccm and 50 sccm for MoS_2_ fabrication and for WS_2_ and MoS_2_/WS_2_ respectively. The SiO_2_/Si (1 × 1 cm^2^) substrates were rinsed successively in deionized water, acetone, and ethanol in an ultrasonic bath for 10 min each.

A powder consisting of WO_3_ (≥99.8%), MoO_3_ (≥99.8%), or mixed WO_3_/MoO_3_ with ratio 1:1 was mixed with Sulphur ≥99.98% using a ball-milling machine. All chemicals were purchased from Sigma Aldrich (Saint Louis, MO, USA). A diluted suspension solution with a concentration of 100 mg/mL was prepared using either the WO_3_/S, the MoO_3_/S, or the MoO_3_-WO_3_/S with ethanol and subsequently sonicated to enhance the homogeneity of the solution. Prior to the CVD process, a drop of 10 μL of the suspension solution was directly dropped onto the cleaned substrate using a pipette, as shown in Figure 1. Subsequently a 250 mg of sulfur powder was introduced at the edge of the sealed end of the quartz tube. Initially, the furnace was heated from room temperature up to 400 °C at a 20 °C/min heating rate, then to 850 °C at 5 °C/min for MoS_2_ fabrication and 950 °C at 5 °C/min for WS_2_ and MoS_2_/WS_2_. During the growth of either the MoS_2_, the WS_2_, or the Mo_x_W_1-x_S_2_, the temperature was maintained at 850 °C or 950 °C for 30 min. Then, the furnace was allowed to cool down naturally to room temperature.

The morphology of the fabricated specimens was analyzed using a dual beam focused-ion beam and scanning electron microscope (FIB-SEM) Scios 2 ThermoFisher Scientific (Waltham, MA, USA) microscope. The same tool was also used for the preparation of thin lamella for the transmission electron microscopy (TEM) study. TEM study was carried out using Tecnai and Titan systems from ThermoFisher Scientific (Waltham, MA, USA). TEM samples were prepared on a thin carbon coated Cu mesh grid by transferring the grown TMD samples through a gentle physical exfoliation. The vibrational modes of the processed samples were examined with a micro-Raman spectrometer Renishaw (Wotton-under-Edge, UK), using a laser excitation of 532 nm. The crystalline structure was investigated by X-ray diffraction (XRD) using a D8 Discover diffractometer Bruker (Billerica, MA, USA); K_aCu_ = 1.54 Å. The optical properties were investigated using a UV-Vis-near IR spectrometer JASCO V-670. An X-ray photoelectron spectroscopy (XPS) study was carried out using a PHI VersaProbe III scanning XPS microprobe Physical Electronics (Chanhassen, MN, USA), equipped with a monochromatic and microfocused Al K-Alpha X-ray source (1486.6 eV). During the experiment, an E-neutralizer (1 V), was implemented. CasaXPS processing software 2.3. was used for the calibration and the curve fitting. Finally, electrical measurements were performed using Palmsens-4 electrochemical workstation under ambient conditions.

## 3. Results and Discussion

### 3.1. Material Characterization

**Figure 2:**
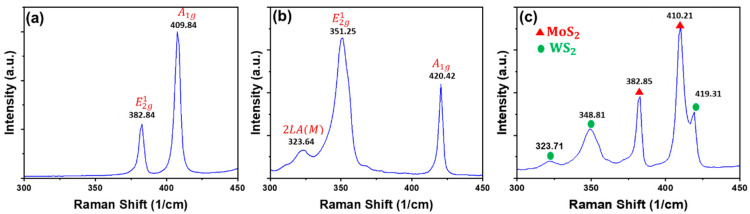
Vibrational modes obtained under 532 nm laser excitation for the (**a**) MoS_2_, (**b**) WS_2_ and (**c**) Mo_x_W_1-x_S_2_ samples.

Figure 2a presents the Raman spectra of the MoS_2_ sample. The observed main Raman vibrational modes indicate the presence of hexagonal 2H-MoS_2_ such as E^1^_2g_ (382 cm^−1^) and A_1g_ (409.8 cm^−1^), which correspond to the in-plane and out-of-plane atomic vibrations, respectively [30].

The main Raman vibration modes recorded for WS_2_ correspond to a 2H-WS_2_ structure such as 2LA(M), E^1^_2g_ and A_1g_ as shown in Figure 2b. The strongest peak at 350 cm^−1^ may be fitted with two sub-peaks with maximum frequencies of 323.6 cm^−1^ and 351.3 cm^−1^ leading to 2LA(M) and E^1^_2g_, respectively. The first-order vibrational mode E^1^_2g_ represents the in-plane vibration between sulfur and tungsten atoms while the A_1g_ vibrational mode at 420 cm^−1^ corresponds to the out-of-plane vibration of sulfur atoms. It is worth noting that the A_1g_ is sensitive to the number of WS_2_ layers [31].

Regarding the Mo_x_W_1-x_S_2_ heterostructure, the Raman peaks corresponding to 2H-MoS_2_ and 2H-WS_2_ are present, as shown in Figure 2c. The positions of the E^1^_2g_ and A_1g_ vibrational modes do not seem to shift compared to the observed peaks in individual samples. This indicates that WS_2_ and MoS_2_, obtained through our preparation route, have no effect on each other’s long-range Coulomb interactions between the effective charges as previously reported [24].

**Figure 3:**
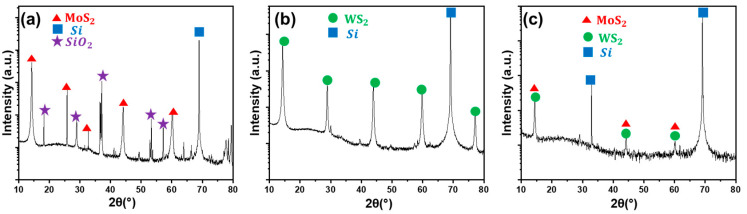
XRD diagrams recorded for the as-grown (**a**) MoS_2_, (**b**) WS_2_ and (**c**) Mo_x_W_1-x_S_2_.

The x-ray diffraction (XRD) diagram illustrated in Figure 3a shows clear diffraction peaks at 14.25°, 25.81°, 32.15°, 44.13° and 60.21° corresponding to 2H-MoS_2_. They are attributed, respectively, to the (002), (004), (103), (006) and (008) planes of the hexagonal 2H-MoS_2_. For WS_2_, the XRD diagram shows several significant diffraction peaks at 14.3°, 28.8°, 43.9°, 59.8°, and 77.13° as can be seen in Figure 3b. They are attributed to 2H-WS_2_ planes (002), (004), (006), (008) and (00,10).

For the Mo_x_W_1-x_S_2_ heterostructure, the MoS_2_ and WS_2_ peaks are present in the corresponding XRD diagram given in Figure 3c. This confirms the successful fabrication of the heterostructure. The sharp diffraction peaks observed on the spectra are a clear indication of the high crystallinity of the fabricated nanosheets.

**Figure 4:**
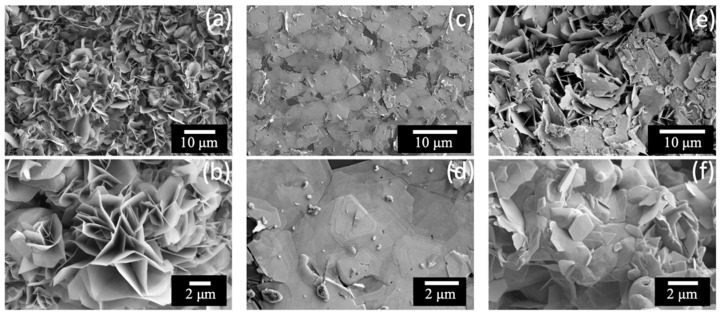
Low and high magnification SEM images of (**a**,**b**) MoS_2_ vertically aligned nanosheets (**c**,**d**) WS_2_ stacked layers and (**e**,**f**) Mo_x_W_1-x_S_2_ co-existent vertical and stacked layers.

The SEM images show different morphologies for the MoS_2_, WS_2_ and the heterostructure samples. The MoS_2_ flakes (Figure 4a) are observed to grow vertically. This is highlighted at a higher magnification in Figure 4b. Similar results were reported previously [32]. On the other hand, WS_2_ shows accumulated crystals stacked on top of the substrate. A large number of triangular shaped flakes disposed horizontally are visible in Figure 4c,d at low and high magnifications, respectively.

As can be seen in Figure 4e,f, the Mo_x_W_1-x_S_2_ heterostructure exhibits a mixed morphology. It consists of both vertically aligned MoS_2_ nanosheets and stacked layers of WS_2_. A coherence between the two phases is observed with no visible segregation between the two compounds.

The change in the MoS_2_ and WS_2_ morphology could be attributed to the following hypotheses: (1) The high CVD reaction temperature used to process the WS_2_ could enhance the nucleation kinetics of the first WS_2_ seeds allowing the coalescence process to occur horizontally. In contrast, for MoS_2_ the reaction temperature is lower leading to dispersed seeds in the surface of the substrate favoring the coalescence on the top of the first layers; (2) The WS_2_ weight may impede the vertical shape of WS_2_; (3) The flow rate used for the processing of WS_2_ (50 sscm) is lower compared to the one for MoS_2_ fabrication (70 sccm), which may not allow the evacuation of sulfur excess.

**Figure 5:**
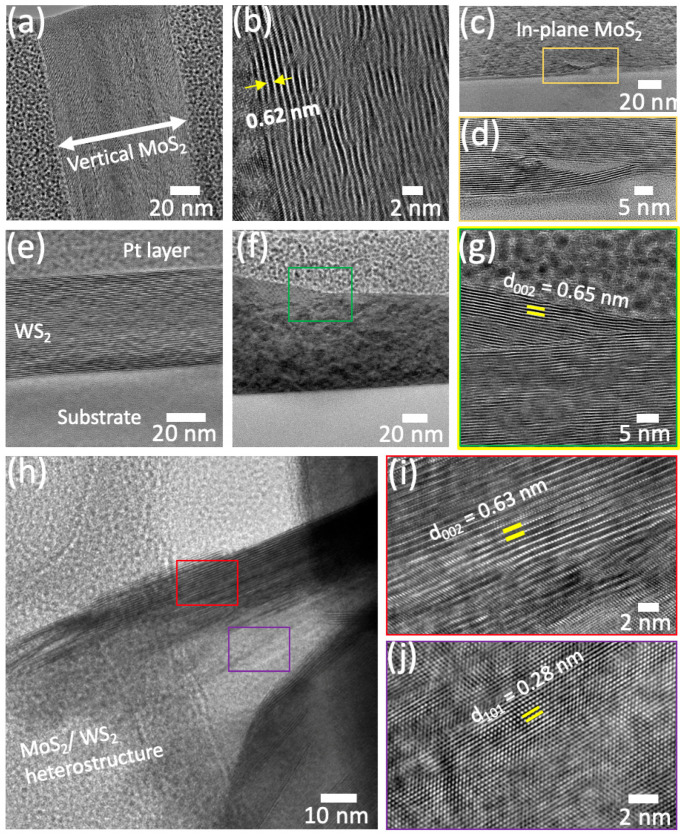
Typical TEM microstructure at low and high magnifications. (**a**,**b**) Cross-sectional bright field TEM images of vertically oriented MoS_2_ flakes; (**c**,**d**) TEM image of in-plane MoS_2_; (**e**–**g**) cross-sectional views of deposited multi-stacked WS_2_ layers; (**h**–**j**) bright-field image of the Mo_x_W_1-x_S_2_ heterostructure.

In order to comprehend the mixing mechanism of MoS_2_ and WS_2_ structures, we have conducted further microstructure analysis using HRTEM for the three samples as shown in Figure 5.

Figure 5a shows a TEM cross-sectional view of the vertically oriented MoS_2_ nanosheets (thickness ~100 nm). Higher resolution imaging (Figure 5b) indicates an interplanar spacing of 2H-MoS_2_ of ~0.62 nm. Moreover, Figure 5c depicts the base region at the interface between the MoS_2_ and the substrate, showing the nucleation of the 2H-MoS_2_. The zoomed view in Figure 5c indicates the nature of the flakes’ growth, where certain layers tend to be continuous and few sheets get terminated due to the absence of growth space, which could be at the origin of the vertically aligned 2H-MoS_2_. Figure 5e–g shows the cross-sectional views of the WS_2_ sample. The smooth planar growth of the WS_2_ is clearly visible compared to MoS_2_, in agreement with the observation on the SEM images. An interplanar distance of 2H-WS_2_ is determined at ~0.65 nm. Figure 5h shows typical TEM bright field images obtained from the Mo_x_W_1-x_S_2_ heterostructure showing an overlapping region (red and purple boxes). Zoomed images of both boxes indicate the presence of 2H-MoS_2_ (red box, Figure 5i) and 2H-WS_2_ (purple box, Figure 5j), which is a signature of the MoS_2_-WS_2_ heterostructure form with a crystallographic relationship (200)_MoS2_//(101)_WS2_. Moreover, it is worth noting that 2D materials in their single-phase form are often considered to be thermally stable. Nevertheless, in their heterostructure form, they may suffer from thermal defects and induced stresses that could affect their electronic and optical properties. In the current work, these inherent stresses have not been evaluated as well as their impact on the heterostructure physical properties. It is believed that the effects of these stresses on the heterostructure structural and electronic properties cannot be neglected.

**Figure 6:**
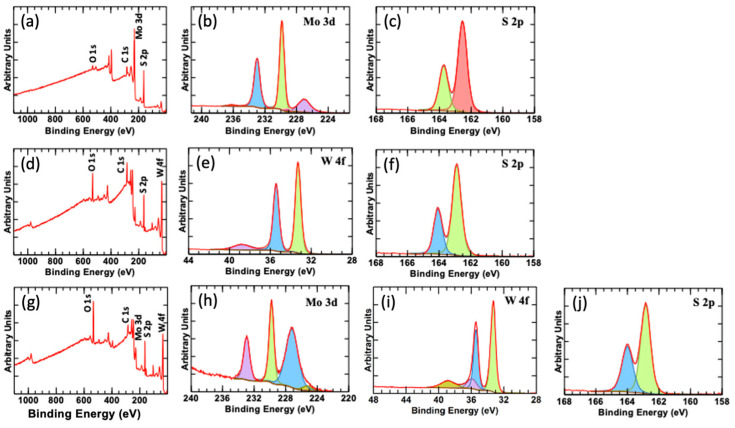
XPS analyses for the (**a**–**c**) MoS_2_, (**d**–**f**) WS_2_ and (**g**–**j**) Mo_x_W_1-x_S_2_ samples.

To precisely investigate the chemical composition of the fabricated samples, we have conducted XPS analyses, as shown in Figure 6.

The XPS survey scan of the MoS_2_ sample, shown in Figure 6a, indicates the presence of MoS_2_ constituting elements. This figure shows strong peaks for Mo 3d and S 2p orbitals. The peak at 227.02 eV corresponds to the S 2s peak. The two strong peaks at 229.85 eV and 232.99 eV are attributed to Mo^4+^ 3d_5/2_ and 3d_3/2_ (Figure 6b). A small peak appearing at 236.2 eV indicates a minor oxidation of the Mo material [33]. For Sulfur, S 2p peaks are recorded and shown in Figure 6c. The two strong peaks at 162.54 eV and 163.73 eV are attributed to S^2−^ 2p_3/2_ and p_1/2_ states. Regarding the WS_2_, the XPS survey scan (Figure 6d) indicates the presence of W and S in the material, translated by the strong peaks for W 4f_7/2_ and W 4f_5/2_ appearing at 33.28 eV and 35.43 eV, respectively (Figure 6e). An additional peak appears at 38.72 eV that could be attributed to W p_3/2_. The S peaks appearing at 162.88 eV and 164.07 eV are due to the S^2−^ 2p_3/2_ and p_1/2_ states, respectively (Figure 6f). Moreover, the XPS survey scan obtained from the Mo_x_W_1-x_S_2_ heterostructure sample is provided in Figure 6g, indicating the presence of W, Mo, and S in the heterostructure. The Mo 3d peaks are found at 229.79 eV and 232.95 eV (Figure 6h), and the S 2s peak is found at 227.2 eV. Additionally, strong peaks of W appear at 33.3 eV and 35.42 eV, corresponding to W 4f_7/2_ and W 4f_5/2_. The peak at 35.91 eV could be attributed to W-O bond, while the 38.80 eV peak is attributed to W 5p_3/2_ (Figure 6i). The S peaks appearing at 162.84 eV and 164.0 eV correspond to the S^2−^ 2p_3/2_ and p_1/2_ states, respectively (Figure 6j).

### 3.2. Optical Properties

Density functional theory (DFT) calculations, using generalized gradient approximation (GGA) and Perdew Burke Ernzerhof (PBE) methods (implemented in Quantum Espresso) were first used to estimate the structural optimizations and electronic attributes (see Supplementary Materials). We have summarized in Table 1 the crystal structures of MoS_2_ and WS_2_, as well as the cell parameters, the cutoff wave function and charge densities for both materials implemented in DFT calculations. For all configurations (monolayer and bilayer), the cell parameters and atomic locations were fully relaxed using the Broyden-Fletcher-Goldfarb-Shanno (BFGS) approach until the remaining force on each atom was less than 10^−3^ Ryd/Bohr (see Supplementary Materials).

**Figure 7:**
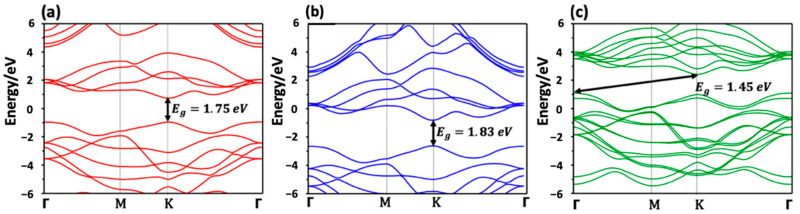
DFT computed electronic bands structure for monolayer (**a**) MoS_2_, (**b**) WS_2_ and (**c**) Mo_x_W_1-x_S_2_.

To simulate monolayers in our calculations, a vacuum space of 15 Å was created along both sides of the z-axis to isolate the crystal and prevent interactions between the adjacent layers. For the sampling of the Brillouin zone, a Monkhorst-Pack technique is used, with k-point meshes of 9 × 9 × 2 for the bulk and 9 × 9 × 1 for the monolayer and bilayer structures. The generated optimal structure was utilized to compute the band structures for various setups. The results revealed a transition from an indirect bandgap to a direct bandgap as the structure changed from bulk to monolayer for both WS_2_ and MoS_2_. Similar results have been reported [29,34,35,36]. The band structure of the MoS_2_ and WS_2_ layers were well conserved, as seen in Figure 7.

The conduction band minimum (CB_min_) and the valence band maximum (VB_min_) of the MoS_2_ and WS_2_ monolayers are positioned at the K point, respectively. Mo_x_W_1-x_S_2_ is an indirect semiconductor with a 1.45 eV indirect bandgap. Unlike their homogeneous bilayers’ counterparts, the CB_min_ of Mo_x_W_1-x_S_2_ heterostructures is positioned at the K point, whilst the VB_min_ is located at the point Γ. Through van der Waals interactions, the MoS_2_ and WS_2_ monolayers produce an atomically sharp type-II heterointerface, which may be favorable for electron–hole pair separation. Free electrons and holes will spontaneously separate in a type II heterostructure, which is useful for optoelectronics and solar energy conversion applications [37,38,39].

**Figure 8:**
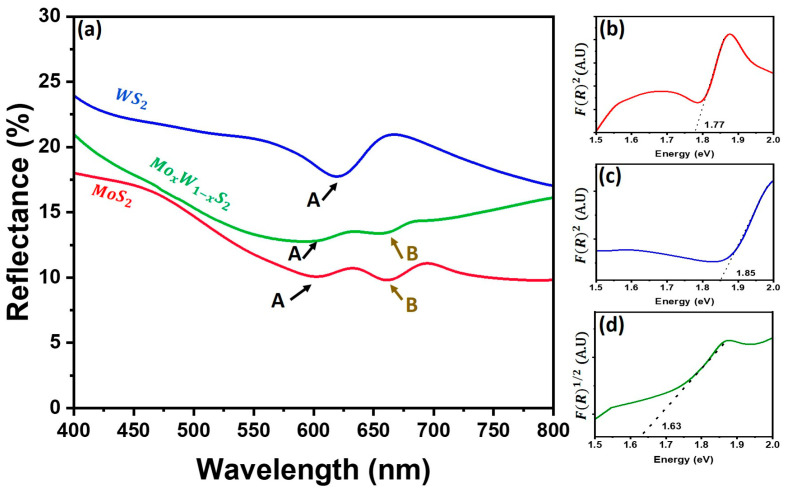
Reflectance for all samples (**a**) (red: MoS_2_; blue: WS_2_ and green: Mo_x_W_1-x_S_2_). The insets (**b**–**d**) show the variation of the reflectance function versus energy determined by the Kubelka-Munk model. The respective bandgap energies are indicated by dashed lines.

Furthermore, the optical reflectance of all samples was measured at room temperature in the wavelength range 400–800 nm as shown in Figure 8a.

MoS_2_ shows the lowest reflectance compared to the other samples, with the presence of both excitons, A and B, clearly visible at 636 nm and 688 nm positions, respectively [40,41]. This is due to the high optical absorption of the vertical morphology of the MoS_2_ nanosheets with high specific area and light trapping via the multiple scattering effects [42]. On the other hand, WS_2_ exciton appears clearly at 620 nm, showing the highest reflectance caused by its planar morphology. Finally, the reflectance of the Mo_x_W_1-x_S_2_ sample shows a mixed behavior between MoS_2_ and WS_2_ with an enhancement of the MoS_2_ excitons. This validates the successful fabrication of the heterostructure as confirmed by the Raman spectroscopy and HRTEM analyses discussed earlier.

To obtain the optical bandgap, the reflectance measurements recorded for all investigated samples are implemented in Kubelka-Munk model as per the following equation [43]:

(1) $$F\left(R\right)=\frac{K}{S}=\frac{{\left(1-R\right)}^{2}}{2R}$$

where *K* represents the molar absorption coefficient, *S* is the scattering factor, and *R* is the reflectance. Our results show that both MoS_2_ and WS_2_ exhibit a bandgap of 1.77 eV and 1.85 eV, respectively, approximately equivalent to bandgap obtained using DFT calculations. In contrast, the Mo_x_W_1-x_S_2_ sample shows a low bandgap of 1.63 eV compared to DFT calculations, which is probably due to the heterostructure construction and implementation in DFT that does not seem to reproduce the effective form of the heterostructure.

### 3.3. Photoresponse Measurements

**Figure 9:**
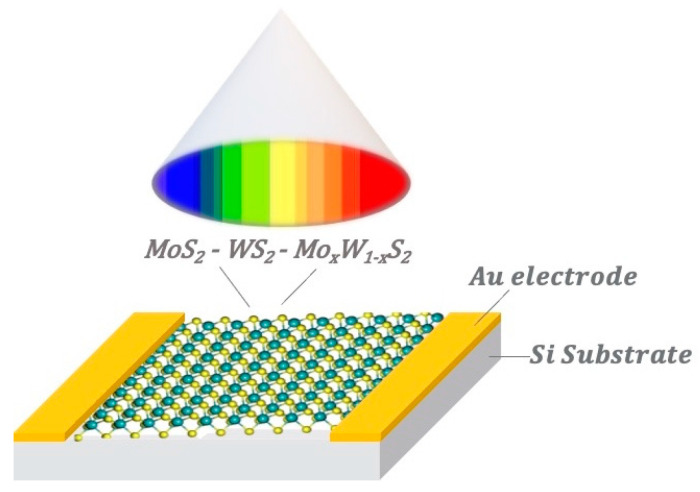
Schematic of the photoresponse measurements set up used for all samples.

To evaluate the optoelectronic properties of the MoS_2_, WS_2_, and Mo_x_W_1-x_S_2_ nanocomposite films, we deposited a pair of Au electrodes onto the device surface, as illustrated in Figure 9. The electrical measurements were conducted at room temperature under dark and illumination conditions using a halogen lamp (70 mW/cm^2^), and under different excitation wavelengths ranging from 400 nm to 700 nm. The effective detection area of the samples was 0.075 cm^2^.

The J-V curves were collected using a voltage sweep program −/+ 5 V at 0.1 V step. The *J_Ph_* at 5 V bias was subsequently computed using the following formula:

(2) $$J_{Ph}\;\left(mA/cm^{2}\right)=\frac{I_{light}-I_{dark}}{A}$$

where *I_light_* and *I_dark_* represent the current obtained in the light and the dark conditions, respectively. A is the active detection area.

The photoresponse (*P*) of our samples was computed using the following equation:

(3) $$P\left(\%\right)=100\frac{I_{light}-I_{dark}}{I_{dark}}$$

The responsivity (*R_λ_*) and the relative detectivity (D*) [17] of the photodetectors were obtained using the following equations:

(4) $$R_{\lambda }=\frac{I_{ph}}{P_{light}}$$

(5) $$D^{*}=\frac{R_{\lambda }}{{\left(2qI_{dark}\right)}^{\frac{1}{2}}}$$

where *q* is the absolute value of an electron charge (1.6 × 10^−19^ Coulombs), *R_λ_* is the responsivity given in units of mA W^−1^, and D* is the relative detectivity given in units of Jones.

**Figure 10:**
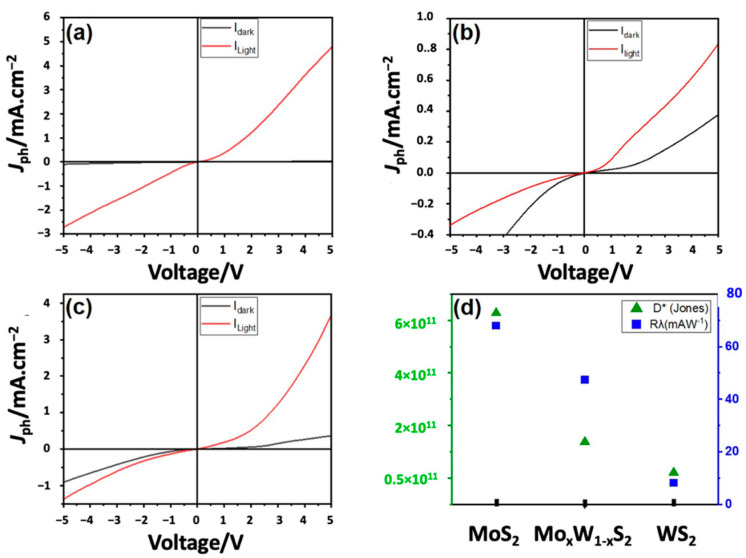
(**a**) Photocurrent density measurements for (**a**) MoS_2_, (**b**) WS_2_, (**c**) Mo_x_W_1-x_S_2_, and their respective (**d**) responsivity and relative detectivity obtained under halogen lamp illumination (70 mW/cm^2^).

In Figure 10, compared to the other samples, MoS_2_ exhibits the highest *J_Ph_* achieving 4.8 mA/cm^2^, compared to the other samples, while WS_2_ and the heterostructure Mo_x_W_1-x_S_2_ have shown lower values of 0.8 mA/cm^2^ and 3.7 mA/cm^2^, respectively. Moreover, the highest photoresponse is also achieved by MoS_2_ ~ $6.8\times 10^{4}$%, while WS_2_ exhibits the lowest one of 1.5 × 10^3^%. The photoresponse of Mo_x_W_1-x_S_2_ heterostructure of ~5.8 × 10^3^% is similar to previously reported values [44]. This strong photoresponse is due to the high optical absorbance of the vertical MoS_2_ nanosheets, known for possessing a high ability to capture light and a quick charge transfer [45] (e.g., Figure 10a–c).

For MoS_2_, we obtain maximum values of *R_λ_* and D*, respectively at 68 mA W^−1^ and 6.3 × 10^3^ Jones, with a 5 V bias voltage. It is worth noting that higher *R_λ_* values were also reported [18,19,46,47], however in those works the considered active area was extremely smaller (~10^−7^ cm^2^) and a high applied bias was considerably higher (~50 V) compared to our present study. Instead, our findings concur that WS_2_ and Mo_x_W_1-x_S_2_ heterostructure exhibit *R_λ_* and D* of 8.9 mA W^−1^, 2.1 × 10^10^ Jones and 47.4 mA W^−1^, 1.4 × 10^11^ Jones, respectively compared to MoS_2_.

**Figure 11:**
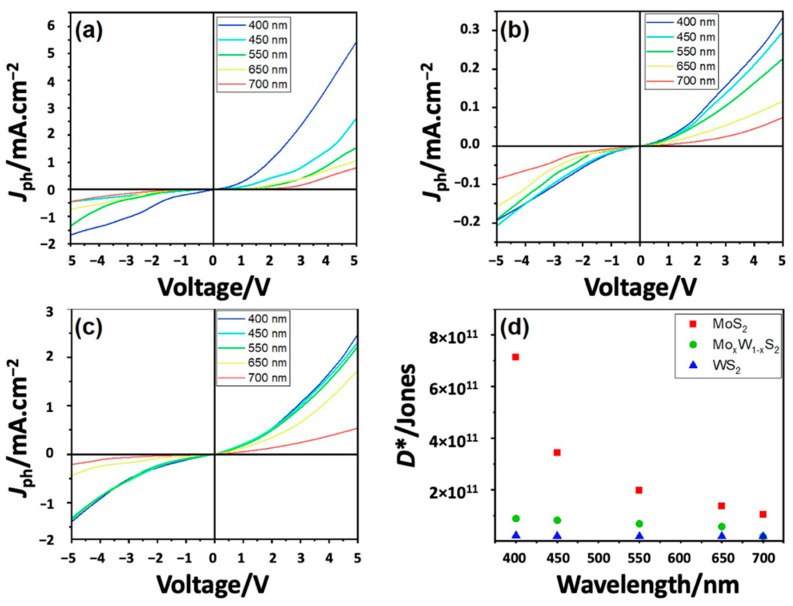
Photocurrent density *J_ph_* measured for (**a**) MoS_2_, (**b**) WS_2_, (**c**) Mo_x_W_1-x_S_2_, and (**d**) their respective relative detectivity under 400–700 nm wavelength excitations.

For further examinations of the photoresponse of our samples, we conducted a series of photoresponse measurements under monochromatic light excitations in the range between 400 nm and 700 nm wavelengths. Figure 11d shows the relative detectivity D* computed at various wavelengths using the above-mentioned formula (Equations (4) and (5)), which is in agreement with the measured values in Figure 10d. From the latter, one can notice that *R_λ_* and D* are decreasing from 77.2 to 10.9 mA W^−1^ and from 7.2 × 10^11^ to 1.8 × 10^10^ Jones, respectively, with an increasing excitation wavelength from 400 nm to 700 nm.

The maximum responsivity *R_λ_* of 77.2 mA W^−1^ and the relative detectivity D* of 7.2 × 10^11^ Jones, were obtained at 400 nm excitation for MoS_2_ sample as reported elsewhere [18,19,46,47].

To further correlate our investigation with existing works, we conducted a survey of available data for sole MoS_2_, WS_2_ and the heterostructure made out of these compounds. The survey is summarized in Table 2.

The photodetection obtained for MoS_2_ shows similar performances in terms of detectivity with slightly better performance of our CVD-fabricated MoS_2_ compared to the sample processed by PLD. However, there is a difference in responsivity, mainly attributed to applied high bias voltages (~50 V) and very low active area used 10^−7^ cm^2^, compared to our active area of 10^−2^ cm^2^. This shows that our one-step fabricated MoS_2_ has exhibited high photodetection performances despite the larger effective area used. To the best of our knowledge, no previous results were reported on WS_2_ better than our results on CVD-grown samples achieving a responsivity of 11.8 mA W^−1^ and a detectivity of 10^10^ Jones, obtained at a very low bias voltage of 5 V. The control of CVD parameters to grow a high quality Mo_x_W_1-x_S_2_ monolayer was already reported [28]. Similarly, we have fabricated the Mo_x_W_1-x_S_2_ using a one-step CVD fabrication method achieving a responsivity and a detectivity of 47 mA W^−1^ and 10^11^ Jones, respectively. Similar results were reported before using a mixture of MoS_2_/WS_2_ monolayers and graphene or using MoS_2_ core shell containing WS_2_. We believe that our results indicate that our processing route consisting of a one-step and low-cost CVD fabrication technique of MoS_2_, WS_2_ and Mo_x_W_1-x_S_2_ hold a strong promising potential for the future development of scalable photodetector devices.

## 4. Conclusions

A Mo_x_W_1-x_S_2_ heterostructure was successfully synthesized by a one-step CVD route. The high purity and high quality of the samples have been confirmed by multiple characterization techniques. The incorporation of the MoS_2_, WS_2_ and the Mo_x_W_1-x_S_2_ heterostructure into photoconductive devices demonstrated a promising potential of these compounds to be used as broadband photodetectors. In particular, the Mo_x_W_1-x_S_2_ heterostructure has achieved a responsivity of 47.4 mA W^−1^ and a relative detectivity of 1.4 × 10^11^ Jones under visible light excitation ranging from 400 to 700 nm.

## Figures and Tables

**Table 1:** Crystal systems, cut off wave function and cell parameters implemented in DFT calculations.

Material	Crystal System	Cut Off/ Wave Function (Ryd)	Lattice Parameters (Å)
MoS_2_	P63/mmc	70/700	a = b = 3.18; c = 15
WS_2_	P63/mmc	50/500	a = b = 3.19; c = 15
Mo_x_W_1-x_S_2_	P63/mmc	60/600	a = b = 3.18; c = 15

**Table 2:** Photodetection performances of our samples with respect to the literature.

Material	Fabrication	Bias (V)	Power Density (mW cm^−2^)	Active Area (cm^2^)	Excitation	Responsivity	Detectivity (Jones)	Ref.
					(nm)	(mA W^−1^)		
MoS_2_	PLD	10	8	1.2 × 10^−3^	445–2717	50.7	1.55 × 10^9^	[18]
MoS_2_-HfO_2_	Exfoliation	5	-	1.5 × 10^−7^	550–800	104	7.7 × 10^11^	[45]
MoS_2_/GaN substrate	CVD	20	2.9	4.7 × 10^−4^	460	25 × 10^3^	5.6 × 10^8^	[47]
MoS_2_/Graphene	CVD	10	1	6 × 10^−6^	532–633	1.4 × 10^3^	8.7 × 10^14^	[48]
MoS_2_	CVD	50	7	6.8 × 10^−7^	450–750	105	9.4 × 10^12^	[19]
MoS_2_	CVD	5	70	7.5 × 10^−2^	400–700	77.2	7.2 × 10^11^	[This study]
WS_2_	Sputtering	10	14.9	9 × 10^−7^	450–635	0.4	4.4 × 10^6^	[49]
WS_2_	Sputtering	5	-	-	365	53.3 × 10^3^	1.22 × 10^11^	[50]
WS_2_-Graphene	CVD	5	2.5 × 10^7^	4 × 10^−12^	532	3.5 × 10^3^	1.6 × 10^10^	[23]
WS_2_	Exfoliation	5	11.7	-	532–1064	4.1	2.6 × 10^9^	[22]
WS_2_	CVD	10	0.07	1.7 × 10^−6^	532	0.5	4.9 × 10^9^	[51]
WS_2_	CVD	5	70	7.5 × 10^−2^	400–700	11.8	2.9 × 10^10^	[This study]
MoS_2_/WS_2_ Graphene	CVD	10	1.7 × 10^2^	3.1 × 10^−8^	532	2340 × 10^3^	4.1 × 10^11^	[44]
MoS_2_/WS_2_	2-steps CVD	4	-	1.2 × 10^−5^	450	2.3 × 10^3^	-	[24]
WS_2_/MoS_2_	2-steps CVD	5	1.3 × 10^3^	6.2 × 10^−7^	457–671	6.7 × 10^3^	3.1 × 10^13^	[25]
Mo_x_W_1-x_S_2_	CVD	5	70	7.5 × 10^−2^	400–700	47.4	1.4 × 10^11^	[This study]

## Data Availability

Data would be made available upon request to the corresponding author.

## Supplementary Materials

The following supporting information can be downloaded at: https://www.mdpi.com/article/10.3390/nano13010024/s1, Figure S1: Crystals configuration used in DFT for (a) MoS_2_, (b) WS_2_ and (c) Mo_x_W_1-x_S_2_; Figure S2: Bandgap computed by DFT simulations (a) direct bandgap for monolayer MoS_2_, (b) indirect bandgap for bilayer MoS_2_; Figure S3: Bandgap obtained using Kubelka-Munck model (a) direct bandgap for monolayer MoS_2_, (b) indirect bandgap for bilayer MoS_2_; Figure S4: Bandgap computed using DFT simulations(a) direct bandgap for monolayer WS_2_, (b) indirect bandgap for bilayer WS_2_; Figure S5: Bandgap obtained using Kubelka-Munck model (a) direct bandgap for monolayer WS_2_, (b) indirect bandgap for bilayer WS_2_.
