# Supplementary material for: Photodetection Properties of MoS2, WS2 and MoxW1-xS2 Heterostructure: A Comparative Study
Source: Nanomaterials (Basel). 2022 Dec 21;13(1):24. doi: 10.3390/nano13010024 (PMC9824100; doi:10.3390/nano13010024)
Supplement: Supplementary file 1 [file nanomaterials-13-00024-s001.zip › nanomaterials-2087461-supplementary.pdf]

Supplementary Materials

Photodetection Properties of MoS_2, WS_2 and $Mo_xW_{1-x}S_2$ Heterostructure: A Comparative Study

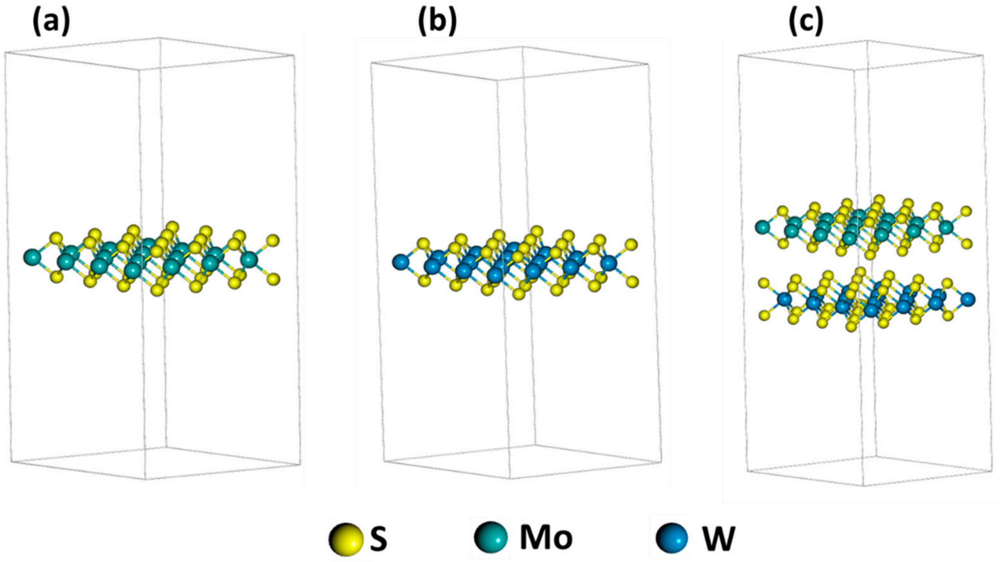

Figure S1: Crystals configuration used in DFT for (a) MoS_2, (b) WS_2 and (c) $Mo_xW_{1-x}S_2$

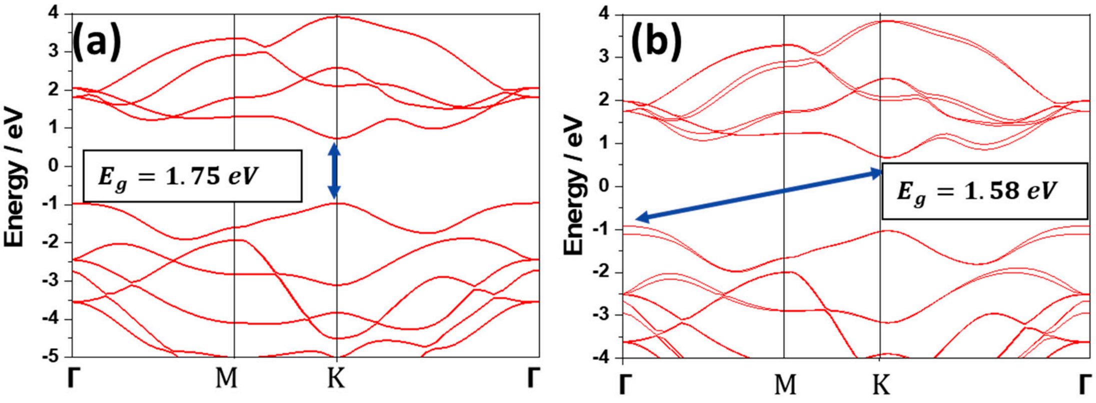

Figure S2: Bandgap computed by DFT simulations (a) direct bandgap for monolayer MoS_2, (b) indirect bandgap for bilayer MoS_2

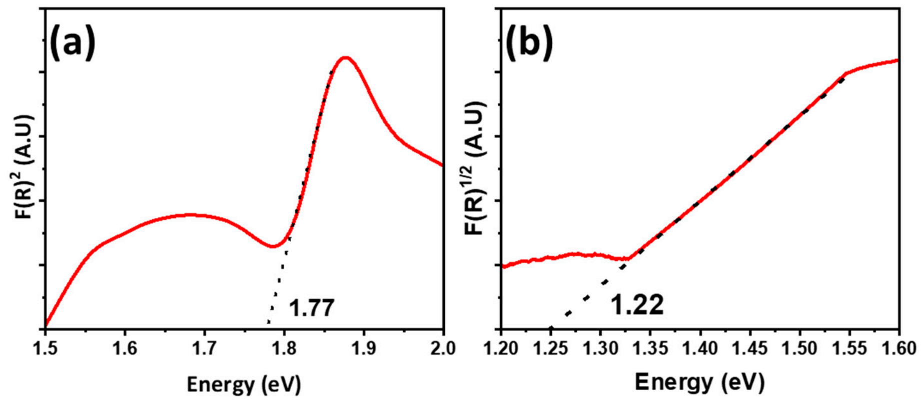

Figure S3: Bandgap obtained using Kubelka-Munck model (a) direct bandgap for monolayer MoS_2, (b) indirect bandgap for bilayer MoS_2

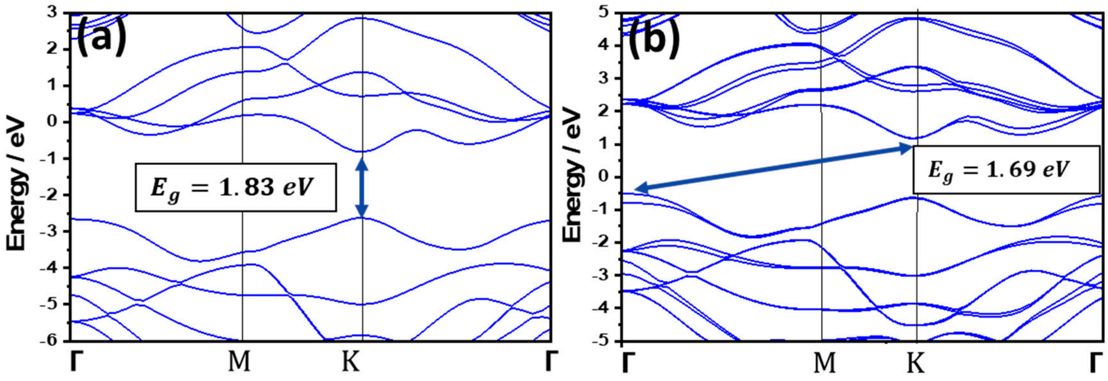

Figure S4: Bandgap computed using DFT simulations(a) direct bandgap for monolayer WS_2, (b) indirect bandgap for bilayer WS_2

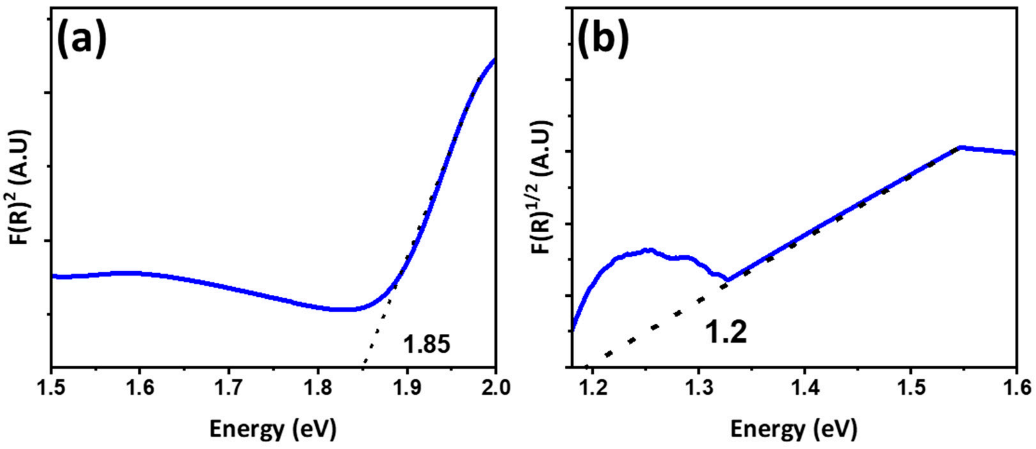

Figure S5: Bandgap obtained using Kubelka-Munck model (a) direct bandgap for monolayer WS_2, (b) indirect bandgap for bilayer WS_2
